# Quantification of circulating cell-free DNA (cfDNA) in urine using a newborn piglet model of asphyxia

**DOI:** 10.1371/journal.pone.0227066

**Published:** 2019-12-31

**Authors:** Polona Rajar, Monica Åsegg-Atneosen, Ola Didrik Saugstad, Rønnaug Solberg, Lars Oliver Baumbusch

**Affiliations:** 1 Department of Pediatric Research, Division of Pediatric and Adolescent Medicine, Oslo University Hospital Rikshospitalet, Oslo, Norway; 2 University of Oslo, Oslo, Norway; 3 Department of Pediatrics, Vestfold Hospital Trust, Tønsberg, Norway; Helsingin Yliopisto, FINLAND

## Abstract

Cell free DNA (cfDNA) in plasma has been described as a potential diagnostic indicator for a variety of clinical conditions, including neonatal hypoxia. Neonatal hypoxia or perinatal asphyxia is a severe medical condition caused by a temporary interruption in oxygen availability during birth. Previously, we have reported temporal changes of cfDNA detected in blood in a newborn piglet model of perinatal asphyxia. However, cfDNA can also be found in other body liquids, opening for a less invasive diagnostic prospective. The objective of this study was to test and establish a reliable method for the isolation and quantification of cfDNA from urine and to explore changes in the quantities of cfDNA using a newborn piglet model of asphyxia. Animals were exposed to hypoxia-reoxygenation (n = 6), hypoxia-reoxygenation + hypothermia (n = 6) or were part of the sham-operated control group (n = 6) and urine samples (n = 18) were collected at 570 minutes post-intervention. Two alternative applications of cfDNA measurement were tested, an indirect method comprising a centrifugation step together with DNA extraction with magnetic beads versus a direct assessment based on two centrifugation steps. CfDNA concentrations were determined by a fluorescent assay using PicoGreen and by qRT-PCR. Genomic (gDNA) and mitochondrial DNA (mtDNA) cfDNA were determined in parallel, taking into account potential differences in the rates of damages caused by oxidative stress. In contrast to previous publications, our results indicate that the direct method is insufficient. Application of the indirect method obtained with the fluorescence assay revealed mean cfDNA levels (SD) of 1.23 (1.76) ng/ml for the hypoxia samples, 4.47 (6.15) ng/ml for the samples exposed to hypoxia + hypothermia and 2.75 (3.62) ng/ml for the control animals. The mean cfDNA levels in piglets exposed to hypoxia + hypothermia revealed significantly higher cfDNA amounts compared to mean cfDNA levels in the samples purely exposed to hypoxia (p < 0.05); however, no significant difference could be determined when compared to the control group (p = 0.09). Application of the indirect method by qRT-PCR revealed mean cfDNA levels of mtDNA and gDNA at the detection limit of the technique and thus no reliable statistics could be performed between the observed cfDNA levels in the investigated groups. The methodology for detection and monitoring of cfDNA in urine has to be further optimized before it can be applied in a clinical setting in the future.

## Introduction

A number of publications have reported that an increase in cell free DNA (cfDNA) in blood is a general feature of various conditions and multi-factorial illnesses, including cancer, trauma, stroke and myocardial infarction, burn injuries, sepsis and autoimmune diseases [1–5]. Perinatal asphyxia is an attractive model for investigating the diverse changes of cfDNA. Perinatal asphyxia is caused by limited blood flow or distribution of oxygen to the fetus or infant immediately before, during or after birth leading to hypoxic and/or ischemic events [6]. The lack of oxygen can damage multiple organs, most dramatic the brain. A metabolic shift, provoked by the oxygen deficiency, may cause a cascade of derangements, like decrease of ATP formation, hypoxanthine and lactate accumulation, decreased pH and increased production of reactive oxygen species (ROS) [7]. ROS damages various structures of the mitochondria, including lipid membranes and mitochondrial DNA (mtDNA), and mitochondrial dysfunction has been frequently linked to several diseases, such as cancer and neurological diseases [8,9]. However, ROS damages not only mtDNA, it has also a mutational effect on genomic DNA (gDNA).

Higher concentrations of circulating cfDNA have been observed in premature neonates [10] leading to the hypothesis that cfDNA levels in plasma might reflect hypoxic changes in the brain [11]. Akhter *et al*. (2001) used samples from 16 newborn pigs, exposed to varying degrees of hypoxia, revealing that more low-MW (200–2,000 base pairs (bp)) DNA fragments were present in samples with lower ATP (higher degree of hypoxia) [12]. Using a piglet model of neonatal hypoxia-reoxygenation, we have reported temporal variations in cfDNA concentrations in blood in relation to the intervention procedure [11].

CfDNA was first discovered as circulating DNA in plasma [13]. It is composed of short DNA fragments, usually ranging from 150–250 bp in length. The origin and turn-over of cfDNA in circulation is not completely understood, but processes like ROS generation, cellular breakdown, reparation mechanisms (necrosis, apoptosis and autophagy) and active release (e.g. from tumour cells) seem to be involved [14]. Other sources of cfDNA might be active intra- or extra-cellular release of DNA, leukocyte oxidative burst or extracellular trap formation [15–18]. Remarkably, cfDNA is not only present in blood, it has been found in other body fluids, including cerebrospinal fluid (CSF) and urine [11,19,20]. Measuring cfDNA in urine has several advantages in comparison to the quantification in blood, due to the larger amounts, higher availability, fewer disturbing particles and reduced invasiveness. The detection of DNA fragments in urine is a promising clinical approach, as shown for DNA sequences of HIV viruses, *Mycobacterium tuberculosis*, malaria *Plasmodium* and *Leishmania*, which can be detected in urine samples of infected patients [13]. CfDNA concentrations in urine may originate from different sources: a trans-renal DNA (Tr-DNA) fraction, filtered through the kidney barrier, and a much smaller portion produced by epithelium cells lining the urogenital tract, microorganisms and possible malign or benign tumors in this area [13]. DNA extracted from urine samples is composed of two fractions, high-molecular weight (high-MW) DNA representing whole genome and larger DNA sequences (mostly originating from epithelial cell debris and infectious agents) and shorter, up to 200 bp long fragments, representing the Tr-DNA fraction [13]. When artificially added to plasma and urine samples, short segments of DNA undergo rapid degradation by nucleases, while Tr-DNA shows greater stability as it is slightly protected by histones in the nucleosome structure [13]. Methods for isolating low-molecular weight (low-MW) DNA fraction from urine samples have still not been optimized, as they demonstrate lower efficiency for molecules smaller than 200 bp [21]. Thus, the development of simple and reliable methods for quantification of cfDNA from urine samples could serve as an efficient substitute for detection of plasmatic cfDNA.

### Aim

The aim of this study was to test and establish an assessment protocol for the isolation and quantification of cfDNA in urine from pigs to investigate changes in cfDNA concentrations using a newborn piglet model of perinatal asphyxia.

## Materials and methods

### Ethics approval

The Norwegian Council for Animal Research approved the experimental protocol (approval numbers 4630 and 5723). Animals were handled in accordance with the European Guidelines for the use of experimental animals by researchers certified by the Federation of European Laboratory Animals Science Association (FELASA). It had been confirmed by the Regional Committees for Medical and Health Research Ethics (REC) that an oral consent is sufficient for taking blood and urine samples from a healthy female volunteer, one of the authors, and that further action or permission is not required.

### Sampling and processing of cfDNA in blood and urine

**Fig 1:**
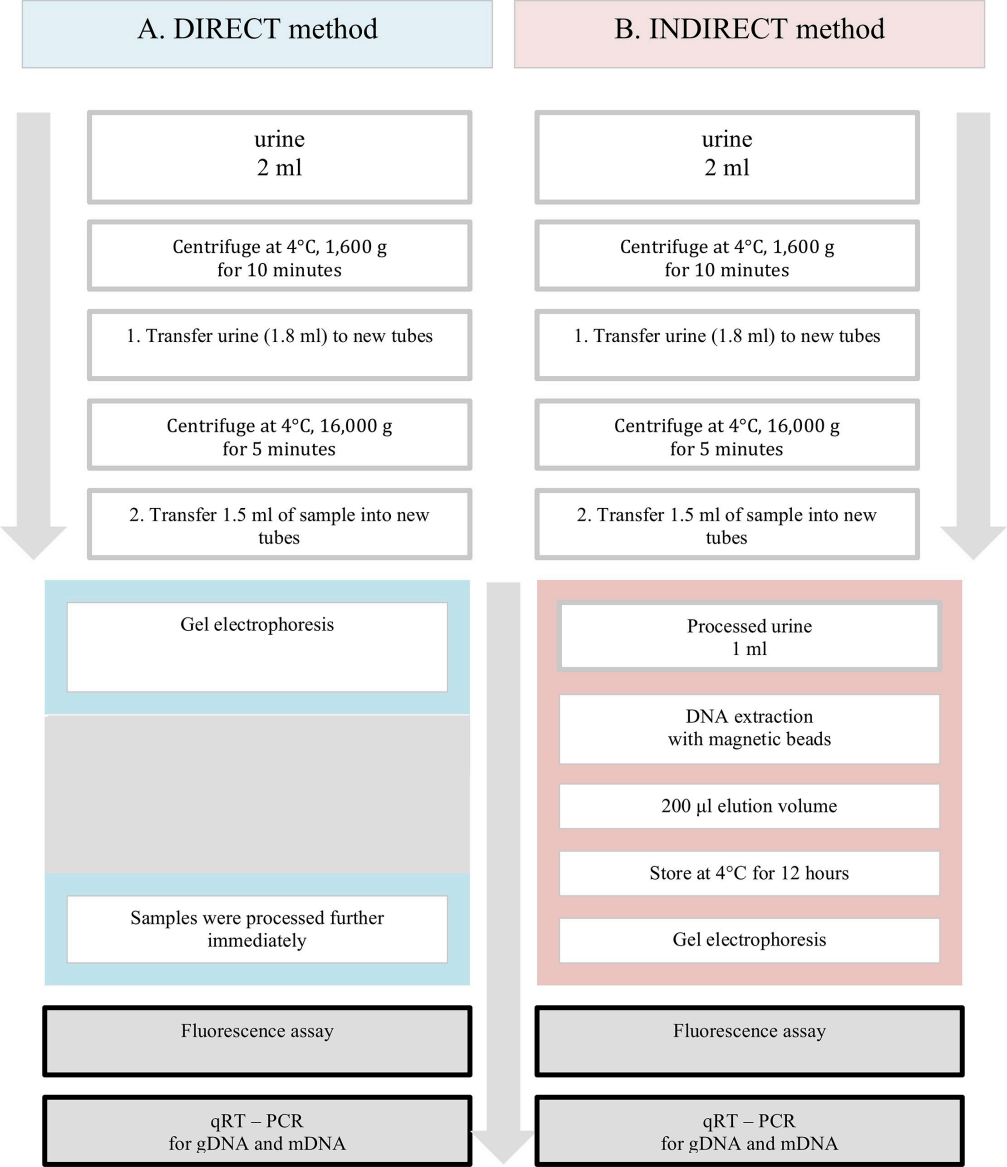
Direct and indirect method for cfDNA quantification. Schematic presentation of the two methods tested for cell free DNA (cfDNA) assessment in urine samples. (A.) A direct method with simply centrifugation and (B.) an indirect method with cfDNA enrichment based on cfDNA extraction using magnetic beads. For cfDNA quantification, all samples were measured by a fluorescent assay and by qRT-PCR, with primers detecting genomic (gDNA) and mitochondrial DNA (mtDNA).

Two essential different approaches were simultaneously tested for cfDNA assessment: A. a direct method and B. an indirect method with cfDNA enrichment. The direct method included only centrifugation steps before DNA quantification, whereas DNA extraction with magnetic beads was used prior to quantification for the indirect method. All samples were processed with both methods in parallels (Fig 1).

Two different quantification techniques were tested and applied for all samples, a fluorescence assay and a qRT-PCR method with primers for both, gDNA and mtDNA (Fig 1).

#### Sampling of blood and urine for method testing

Urine and blood samples from a healthy female individual were used for a pilot experiment. Blood was collected into 4 ml EDTA tubes. Second morning mid-stream urine was sampled into 100 ml plastic containers. Both blood and urine samples were processed immediately by centrifugation at 1,600 g and 4°C for 10 minutes. Approximately 2 ml of plasma (or urine) were transferred into 2 ml sterile tubes and centrifuged again at 16,000 g and 4°C for 5 minutes followed by transfer of 1.5 ml of the supernatant into new sterile tubes. Some of the blood was also processed according to a lymphocyte preparation protocol (Axis-Shield, Oslo, Norway, following the recommendations of the producer), in order to minimize residual white blood cells.

#### Animal samples

**Fig 2:**
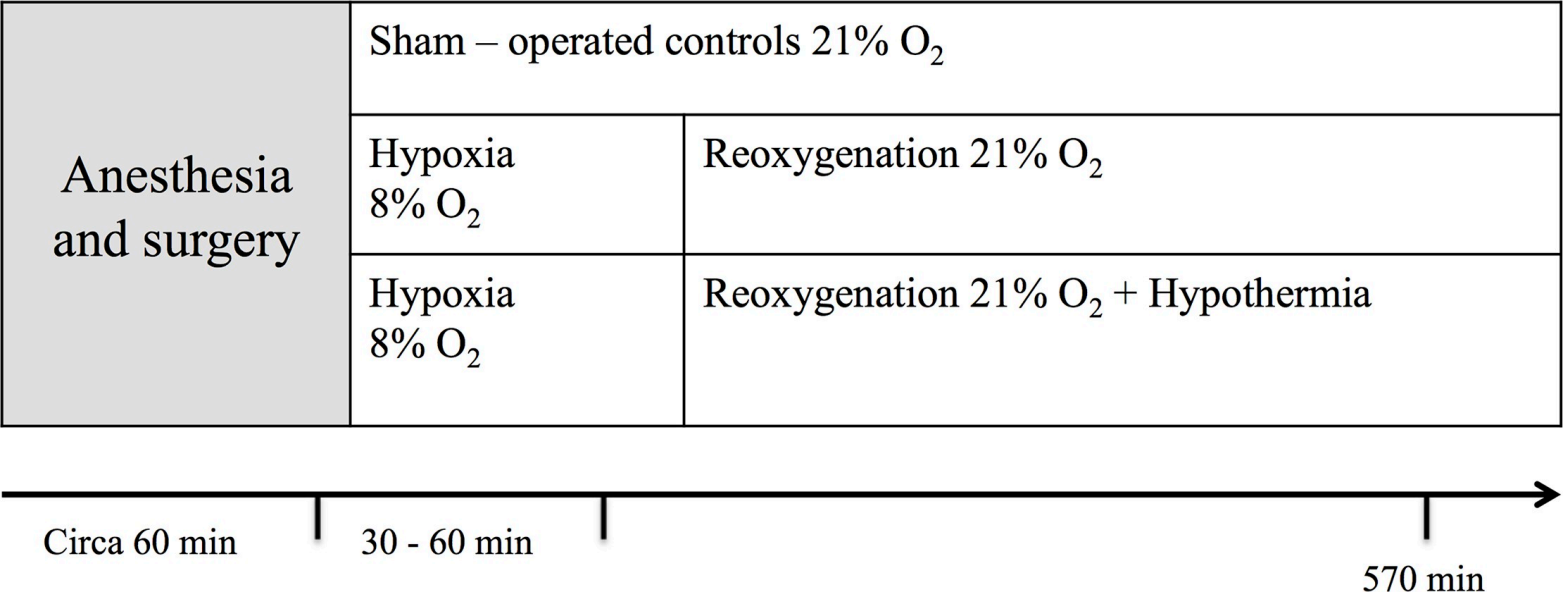
The newborn piglet model of asphyxia exposed to hypoxia-reoxygenation. Newborn piglets were randomized into three study arms: hypoxia-reoxygenation, hypoxia-reoxygenation + hypothermia or sham-operated controls. Perinatal asphyxia was mimicked by breathing 8% O_2_ and CO_2_ aiming at a PaCO_2_ of 8.0–9.5 kPa until a BE of -20 mmol/l and/or mean blood pressure fell below 20 mm Hg. Urine samples were collected at post-intervention, 570 minutes after the end of hypoxia. They were obtained using suprapubic aspiration, transferred to 2 ml tubes and frozen at -80°C.

Piglet samples generated in previous studies build the basis of this animal model of asphyxia [22,23]. The methodological set-up required that at least 2 ml of urine per individual are available for the experiments. Thus, out of the total population of newborn piglets (n = 55), only n = 18 were chosen for the investigations presented here (n = 6 from each group) (Fig 2). All piglets were in good general condition. The animals were anesthetized, ventilated and surgically prepared prior to an exposure to global hypoxemia for 30–60 min and follow-up for another 9.5 h before receiving an intravenous overdose of 150 mg/kg pentobarbital. The control group (n = 6 out of a total population of 11) underwent the same surgical procedures and anaesthesia, with suprapubic urine aspiration as the sole intervention. In both, the hypoxia group (n = 6 out of a total population of 32) and the hypoxia + therapeutic hypothermia group (n = 6 out of a total population of 12), perinatal asphyxia was mimicked by ventilating the piglets with 8% oxygen in nitrogen and adding CO_2_ (aiming at pCO_2_ = 8.0–9.5 kPa) until base excess (BE) reached -20 mmol/l and/or mean arterial blood pressure fell below 20 mm Hg. No further interventions were performed for the hypoxia group. In the hypoxia + hypothermia group, therapeutic hypothermia was induced 30 minutes after exposure to global hypoxemia by cooling piglets on a cooling mattress (Tecotherms TSmed 200; TecCo, Halle, Germany) to 35°C (±0.5°C). The piglets in all other groups were kept at normal body temperature (39°C, measured rectally). Approximately 1–2 ml of urine was collected from each piglet at the age of 12–36 hours with suprapubic thin needle aspiration. Urine samples were immediately snap-frozen in liquid nitrogen and stored in plastic tubes at -80°C (Fig 2).

#### Direct method for animal samples

A minimum amount of 2 ml of urine samples from the piglets was left on the bench at room temperature for 20 minutes to thaw before processed by centrifugation at 1,600 g at 4°C for 10 minutes. Approximately 1.5 ml of urine supernatant were transferred to novel 2 ml sterile tubes and centrifuged again at 16,000 g at a temperature of 4°C for 5 minutes, after which 1.2 ml were transferred into new sterile tubes. After centrifugation the samples immediately were processed for qRT-PCR analysis and fluorescence assay in order to avoid degradation of cfDNA.

#### Magnetic beads-based cfDNA extraction

Samples for cfDNA enrichment were loaded into the King Fisher Duo Prime Purification system (Thermo Fisher Scientific, Waltham, Massachusetts, USA) for magnetic beads DNA extraction following centrifugation to prevent possible gDNA leakage from cell residues. The Mag-Bind cfDNA Kit (Omega Bio-tek, Georgia, USA) was used for extraction of cfDNA from plasma and urine following the recommendations of the producer. Different starting volumes of samples were tested, ranging from 100–2,000 μl. For processing porcine urine, 1,000 μl samples were used and eluted into a final volume of 200 μl. Once the samples were eluted into buffer, they were stored for up to 12 h at 4°C, before they were determined by qRT-PCR and fluorescence quantification.

#### Standard curve

Standard samples with known DNA concentrations were made by diluting either Human Genomic DNA: male (Promega, Madison, Wisconsin, USA) or Porcine DNA stock: female from normal tissue, (Ambisio, Abingdon, UK) with elution buffer. Concentrations in the standard samples were verified using the NanoDrop 1000 instrument (Thermo Fisher Scientific, Waltham, Massachusetts, USA) (S1 Fig). However, due to the detection limit of the method, only DNA standard curve values with concentrations above 1,250 ng/ml could be measured precisely using the NanoDrop 1000 instrument. Since we expected lower concentrations of DNA in our samples, we used fluorescence assay with PicoGreen to measure concentrations in standards diluted to 0.39 ng/ml for both, the fluorescent and the qRT-PCR method (S1 Fig).

### Fluorescence assay for cfDNA quantification

For the PicoGreen fluorescent assay (Quant-iT PicoGreen dsDNA from Molecular Probes, Life technologies, Foster City, USA) PicoGreen dye was diluted 1:200 with elution buffer. For samples processed according to the direct protocol, we used 10 μl of sample diluted with 90 μl elution buffer and added 100 μl diluted PicoGreen. For the indirect protocol, 100 μl of sample and 100 μl diluted PicoGreen were used. The template was kept in the dark and fluorescence immediately measured at an emission wavelength of 485 nm with Victor TM X3 (Perkin Elmer, Waltham, USA).

### Quantitative qRT-PCR reaction and conditions

To cover the various sources and natures of cfDNA, primers were designed for both, gDNA and mtDNA and the possible differences in length of cfDNA products was also taken into account (Table 1). Primer Express 3.0 (Applied Biosystems, Foster By, California, USA) was used to design primers, following the settings recommendations of Applied Biostystems (Tm 58–60°C, GC-content 40–70%, primer length 18–25 bp, <2°C difference between primer pairs, 2-3/5 G or C at the 5’end, maximum of 2/5 G or C at the 3’end, < 4 continuous G, C, A, T, secondary structure was also checked for hairpins or other cross-connections) and the primer efficiency was tested. The qRT-PCR reactions were preformed with an Applied Biosystems Viia7 qRT-PCR instrument (Life technologies, Foster City, USA). Reaction mixtures contained 5 μl DNA standards, sample or elution buffer (for negative control), 2 μl of each primer (10 μM) and 12 μl SYBR Green mix and MQ water to a final volume of 25 μl. The qRT-PCR program was set to initial activation step of 50°C for 2 minutes and 95°C for 10 minutes, followed by 40 cycles of denaturation step at 95°C for 15 s and annealing and elongation at 61°C for 2 minutes. All samples were run in parallels.

**Table 1:** Primers for the qRT-PCR reactions. For quantification of mitochondrial DNA (mtDNA) the NADH6 gene (NADH dehydrogenase subunit 6) was investigated and for genomic DNA (gDNA) quantification, the HK2 gene (hexokinase 2) was examined. To reveal differences in relation to product length, primers for PCR- products with variations in fragment size were designed using the Primer Express 3.0 program from Applied Biosystems.

		Direction: 5’-3’	Tm (°C)	GC (%)	Primer length (bp)	Product length (bp)
**mtDNA**	**NADH6, short product**					
	FP	TCACCCTCAATGACGAACAAGA	59.2	45	22	140
	RP	TAGGGCTCAGGCGTTTGTGTA	59.4	52	21	
	**NADH6, middle-length product**					
	FP	ACAATCGGCATCAACCAACC	59.2	50	20	282
	RP	TAGGGCTCAGGCGTTTGTGT	59.4	52	21	
	**NADH6, long product**					
	FP	ATCGGCCTACTCCTAGCTGCA	60.0	57	21	588
	RP	TAGGGCTCAGGCGTTTGTGTA	59.4	52	21	
**gDNA**	**HK2, short product**					
	FP	GTTCCTGGCTCTGGATCTTGG	59.7	57	21	133
	RP	GCCACTGCCTCGCATGA	59.0	65	17	
	**HK2, long product**					
	FP	GGCTGCAGAATGACCACCAT	59.9	55	20	516
	RP	GTTCCTGGCTCTGGATCTTGG	59.7	57	21	

### Gel electrophoresis

To confirm PCR-product lengths, gel electrophoresis using agarose gels was performed with 1–3% agarose in 0.5 x TBE.

### Statistics

Descriptive statistics, t-test for independent samples and graphs were preformed with IBM SPSS Statistics Version 25 (25.0.0.2) (International Business Machines, Armonk, New York, USA). A power analysis has been performed to address and confirm the feasibility of the methods.

## Results

### Reference values of cfDNA in plasma and urine measured in a healthy human individual

Previous studies have reported in healthy individuals cfDNA concentrations of about 65–877 ng/ml in plasma and 0–215 ng/ml in urine [24,25]. In a pilot study, applying the indirect method with magnetic beads DNA isolation, we detected similar values for cfDNA in blood and urine in samples from a healthy individual (69.18±12.70 ng/ml and 81.19±12.47 ng/ml with fluorescence assay, respectively).

### Levels of cfDNA in urine determined in a newborn piglet model of asphyxia

#### a.) cfDNA concentrations in samples collected with the direct method

**Fig 3:**
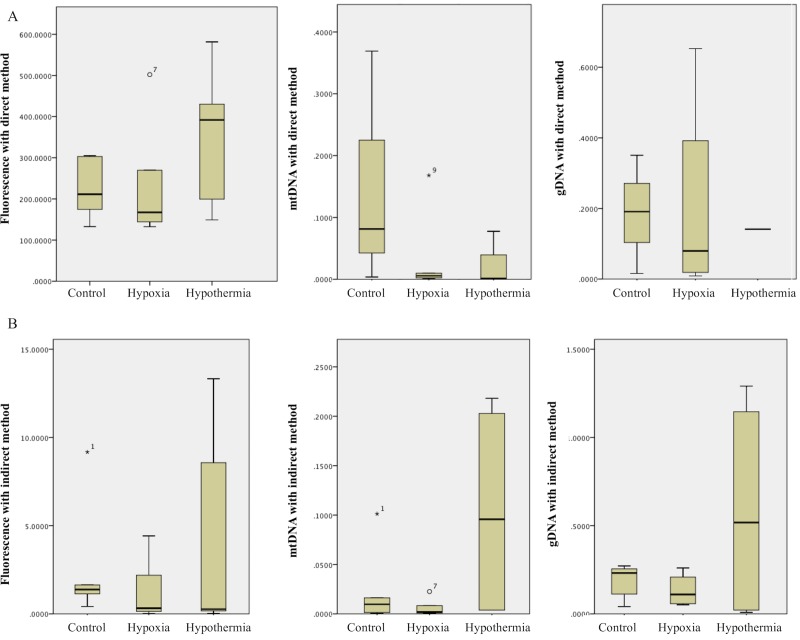
CfDNA concentrations, measured in urine samples of newborn piglets exposed to hypoxia-reoxygenation, processed according to different methods. For quantification of cfDNA in the urine samples, collected using a newborn piglet model of asphyxia with the interventions strategies hypoxia-reoxygenation ("hypoxia"), hypoxia-reoxygenation + hypothermia ("hypothermia") or sham-operated controls ("control"), the following strategies were tested: (A.) CfDNA levels in samples processed according to the direct method. No statistically significant difference between the groups was found. (B.) CfDNA levels in samples measured using the indirect method. When measured with fluorescence, cfDNA concentrations in the hypoxia + hypothermia group were higher compared to cfDNA levels in the hypoxia group, but not when compared to controls (p = 0.09). QRT-PCR investigating mtDNA and gDNA targets, revealed higher cfDNA levels in the hypoxia + hypothermia group when compared to the controls and to the hypoxia group; however, cfDNA values were at the detection limit of the technique.

Mean (SD) cfDNA levels obtained with the fluorescence technique were 230.48 (142.25) ng/ml for the hypoxia group, 357.29 (158.94) ng/ml for the hypoxia + hypothermia group and 233.01 (69.23) ng/ml for the control group, respectively. No statistically significant differences could be found between the cfDNA mean levels in the investigated groups (t-test for independent samples) (Fig 3A).

The cfDNA amounts in these samples were analyzed by qRT-PCR using primers for mtDNA (MTH primers) or gDNA (HK2 primers): mean (SD) cfDNA levels obtained were 0.19 (0.17) ng/ml, 0.03 (0.07) ng/ml and 0.21 (0.3) ng/ml for the hypoxia group, 0.03 (0.04) ng/ml and 0.14 ng/ml (only one valid result) in the hypoxia + hypothermia group and 0.51 (0.92) ng/ml for the control group, respectively. The attained values were at the detection limit of the method and thus no reliable statistics could be performed between the observed cfDNA levels in the investigated groups (Fig 3A).

#### b.) cfDNA levels measured using the indirect method

Mean cfDNA levels (SD) obtained with the fluorescence assay revealed 1.23 (1.76) ng/ml for the hypoxia samples, 4.47 (6.15) ng/ml for the samples exposed to hypoxia + hypothermia and 2.75 (3.62) ng/ml for the control animals. The mean cfDNA levels in piglets exposed to hypoxia + hypothermia showed significantly higher cfDNA amounts compared to mean cfDNA levels in the samples purely exposed to hypoxia (p < 0.05); however, no significant difference could be determined when compared to the control group (p = 0.09).

Investigating the same probes by qRT-PCR for mtDNA or gDNA targets, we found mean cfDNA levels (SD) for the hypoxia group of 0.01 (0.01) ng/ml and 0.13 (0.10) ng/ml, for the hypoxia + hypothermia group 0.10 (0.12) ng/ml and 0.58 (0.66) ng/ml and 0.03 (0.04) ng/ml and 0.19 (0.09) ng/ml for the control group. Here, mean cfDNA levels attained with mtDNA and gDNA primers illustrated higher amounts of cfDNA measured in animals exposed to hypoxia + hypothermia in comparison to the group of piglets purely exposed to hypoxia and when compared to the control group. Applying the qRT-PCR method, cfDNA values were at the detection limit of the technique and thus reliable statistic tests were difficult to perform (Fig 3B). The exact cfDNA levels determined with each method for the different groups are listed in the supplement (S1 Table).

#### c) Calculating mtDNA and gDNA ratios

The ratios between the amounts of mtDNA and gDNA cfDNA were calculated to evaluate possible differences in the effect of hypoxia on the genomic and mitochondrial level. However, no recognizable pattern or statistically significant differences were detected between the therapeutic groups (S2 Table).

## Discussion

### Investigating the quantities of cfDNA in blood and urine

In a clinical setting, urine is used as a diagnostic sample for different clinical conditions, such as diabetes mellitus, urinary tract infections (UTI), pregnancy, renal failure and drug consumption [26]. Urine aliquots can be obtained easily, without risk and in large quantities. In general, investigating cfDNA in urine instead of blood provides several advantages. Thus, it is surprising that cfDNA detection and quantification in urine is somehow underestimated, possibly due to a lack of standard protocols for cfDNA isolation and common low concentrations, expected to account for less reliable results [27]. Another advantageous example of cfDNA extracted from urine is monitoring of tumour mutations in oncology, because taking cfDNA samples is less invasive in comparison to the procedure of taking a tumour biopsy [28]. Further, for obtaining best quality results, up to 4 ml of plasma are needed [29], which can be problematic in fragile patient groups like children. A proposed limitation for urine samples has been that the passing of cfDNA fragments trough the pores of the glomerular membrane was thought of being unlikely; nevertheless, it has been shown that labelled DNA fragments of 160 bp injected in the peritoneum can later be found in the urine, suggesting that size may be less important as the charge of the molecule itself [13,30].

### Methods for cfDNA extraction: Isolation of cfDNA versus direct measurements

The establishment of high-performance methods for extraction of cfDNA is crucial. Different techniques have been presented based on various physical or chemical features of DNA, including column- or magnetic bead-based systems, comparing (among others) QIAamp DNA Blood mini kit from Qiagen, NucleoSpin Kit from Machery-Nagel and MagNA Pure isolation system from Roche Diagnostics. Fleischhacker *et al*. (2011) found that variations of absolute DNA values were mainly caused by the use of different isolation methods, with MagNA Pure isolation system (Roche Diagnostics, Mannheim, Germany) yielding highest DNA concentration [31]. We have previously tested different cfDNA isolation kits for blood samples collected from piglets, including the DNeasy Blood & Tissue Kit from Qiagen, NucleoSpin Plasma XS Kit from Macherey-Nagel, and Wizard Genomic DNA Purification Kit from Promega [11]. All the tested methods resulted in very low amounts of cfDNA and we suggested that a direct method for cfDNA isolation should be applied in the future. Breitbach *et al*. (2014) recommended this approach, they quantified cfDNA directly from plasma to undergo the problem of reduced DNA yield by extraction procedures, revealing a 2.79-fold higher cfDNA concentrations in unpurified plasma in comparison to the eluate of the QIAamp DNA Blood Mini Kit [2].

### The direct method to gain cfDNA in urine with solely centrifugation

Consequently, we pursued a method using paramagnetic beads for isolating DNA from urine samples, resulting in equal higher DNA yield, like highlighted in other publications [32]. Samples extracted by the direct method (two centrifugation steps without any other prior treatment) showed increased higher-MW DNA contamination in comparison to samples extracted using the magnetic beads DNA method, where almost no high- or low-MW DNA was detectable. High-MW DNA was additionally found in samples, in which white blood cells (WBC) had been removed by a lymphoprep protocol. The amount of DNA lost by the applied technique was determined by spiking experiments, adding non-fragmented human DNA (990 μl plasma spiked with 10 μl 1250 ng/ml Human Genomic male DNA (Promega, Madison, USA)). Again, remaining high-MW DNA was detected in samples gained by the direct method (S2 Fig).

QRT-PCR quantification of human plasma samples processed according to the direct method resulted in values below the detection limit, most probably due to PCR-inhibitors left in the plasma. For urine samples, qRT-PCR quantification was successful; however, concentrations obtained with qRT-PCR were at the detection limit, making statistical performance and interpretation of the results difficult.

### The indirect method to receive cfDNA from urine samples using magnetic beads

Nucleic acids in dispersed biological samples like blood, urine, tissue homogenates and cultivation media bind to coated magnetic beads and unwanted cell components causing PCR-inhibitions; thus, cell debris, proteins or carbohydrates can be washed away [33]. Magnetic beads provide further the possibility of up-concentrating low-abundant molecules in a sample, a great advantage for cfDNA determination. The length of captured cfDNA fragments depends on the magnetic beads type [34]. Increasing the sensitivity of the diagnostic procedure allows for lower starting volumes—sufficient starting volume of an urine sample can be as low as 200 μl [32]. Automation is feasible with magnetic beads, eliminating the need for centrifugation and avoiding the risk of cross-contamination [32,33]. Also, eluted samples may be stored, while quantification of cfDNA in samples without previous DNA extraction has to be processed immediately. Su *et al*. (2008) showed that the magnetic beads method was successful in removing high-MW DNA from urine samples, thus enhancing ability to detect K-ras mutations in the urine of colorectal cancer patients [35]. To avoid loss of cfDNA during isolation and extraction procedures, direct cfDNA quantification from urine and plasma samples has been proposed, with fluorescence assay and qRT-PCR quantification [2,25].

### Quantification of cfDNA by fluorescence assay and qRT-PCR

Commonly used procedures for cfDNA quantification include fluorescent staining (SYBR Gold Nucleic Acid Gel Stain and PicoGreen, Invitrogen, Paisley, UK) and qRT-PCR, with fluorescence being faster and cheaper. However, fluorescent staining is not very specific for small fragment DNA, leading to falsely positive higher concentrations in case of contamination of the investigated samples with gDNA. Fluorescence might be a preferable method, if only relative differences are of interest, e.g. samples gathered along a specific timeline (fall or rise of cfDNA after a traumatic event or before- versus after-treatment).

Quantification with qRT-PCR is more specific. In order to distinguish shorter from longer cfDNA fragments, primers for different fragment lengths have been tested. Primers amplifying sequences larger than cfDNA will provide evidence about left over gDNA in a sample, as previously described by others [36]. By simply subtracting concentration of shorter from longer products, one can eliminated left over gDNA. Studies have shown that increases in concentration fragments larger than <300 bp indicates left over gDNA in the samples [37].

It is still a matter of debate, if gDNA or mtDNA are more sensitive for oxidative stress reactions in a cell; therefore, we decided to amplify two different sequences, one for gDNA and one for mtDNA. For amplification of mtDNA sequences, we designed primer sets of three different lengths, 140 bp, 282 bp and 588 bp with all primers pairs having one primer identical. The HK2 gene was selected for quantifying gDNA, due to its up-regulation in hypoxic conditions [38].

### Clinical relevance of cfDNA determination

It has been proposed that cfDNA levels in plasma could reflect hypoxic changes in the brain [11], which was based on previous observations of higher concentrations of circulating cfDNA in premature neonates [10]. However, cfDNA is not only present in blood, it has been found in other body fluids, including cerebrospinal fluid (CSF) and urine [11,19,20].

In this study, therapeutic hypothermia has been used as a neuroprotective strategy. Although promising, the clinical efficacy of hypothermia has not yet been completely understood [39]. Against our original expectations, we observed higher cfDNA levels in hypoxia + hypothermia group compared to both, control and hypoxia groups.

In our model, the piglets were not treated with muscle relaxants. Shivering of skeletal muscles is a frequent physiological response to hypothermia and a possible source of elevated cfDNA levels [40]. We speculate that this effect could explain higher levels of cfDNA in the hypoxia + hypothermia group. On the cellular level, lack of ATP weakens electron transporter pump in the cerebral cortical nuclear membrane, causing elevated intracellular calcium [39], possibly activating calcium-dependant endonucleases fragmenting unprotected DNA. This could be reflected as higher cfDNA levels in plasma and conceptually in urine.

## Conclusions

We have compared methods to isolate and quantify cfDNA from urine from pigs, using samples from a newborn model of asphyxia. It has turned out that the direct method with simply centrifugation is less reliable, because of the presence of high-MW DNA fragments and remaining PCR-inhibitors. The indirect method with cfDNA enrichment based on cfDNA extraction by magnetic beads is better suited for cfDNA assessments from urine from piglets, due to lower reduced amount of high-MW DNA and other components in the final elute, thus providing more reliable qRT-PCR results. However, in the applied model, the total amounts were at the detection limit of the method. Taking together, urine seems a promising body liquid for cfDNA diagnostics. With methodology in place, this could be a reliable, fast and accessible method. More studies of cfDNA in plasma and urine are required to reveal the nature and relationship of cfDNA to oxidative stress and to investigate a potential clinical relevance.

## Limitations

Limitations of the study are the relative small sample number of animals per investigation group, which could influence the statistical analysis of the results. Further, the follow-up time for the animals is rather short, since we have chosen to use samples from an early response model.

## Supporting information

S1 Fig Standard curve. (A.) Complete standard curve measured with fluorescence assay. (B.) Standard curve magnified for higher dilutions.
(PDF)

S2 Fig Gel electrophoresis of spiked and not-spiked samples, processed with direct and indirect method. (A.) 50kb DNA ladder. Samples extracted by the direct method (E.) showed high-MW DNA contamination in comparison to samples extracted using the magnetic beads DNA extraction method (D.), where no high- or low-MW DNA was visible. Spiking the samples, adding non-fragmented human DNA (F.) (990 μl plasma spiked with 10 μl 1,250 ng/ml Human Genomic male DNA (Promega, Madison, USA) to the samples. Again, left over's of high-MW DNA was detected in samples gained by the direct method (C.), and no high- or low-MW DNA was visible in spiked samples processed with indirect method (B.).
(PDF)

S1 Table Descriptive statistics. CfDNA levels in three therapy groups obtained using different methods.
(PDF)

S2 Table Ratios between mtDNA and gDNA. We found no recognizable pattern or statistically significant differences between the groups.
(PDF)
