# Supplementary material for: Quantification of circulating cell-free DNA (cfDNA) in urine using a newborn piglet model of asphyxia
Source: PLoS One. 2019 Dec 31;14(12):e0227066. doi: 10.1371/journal.pone.0227066 (PMC6938324; doi:10.1371/journal.pone.0227066)
Supplement: S1 Fig — (A.) Complete standard curve measured with fluorescence assay. (B.) Standard curve magnified for higher dilutions. (PDF) [file pone.0227066.s001.pdf]

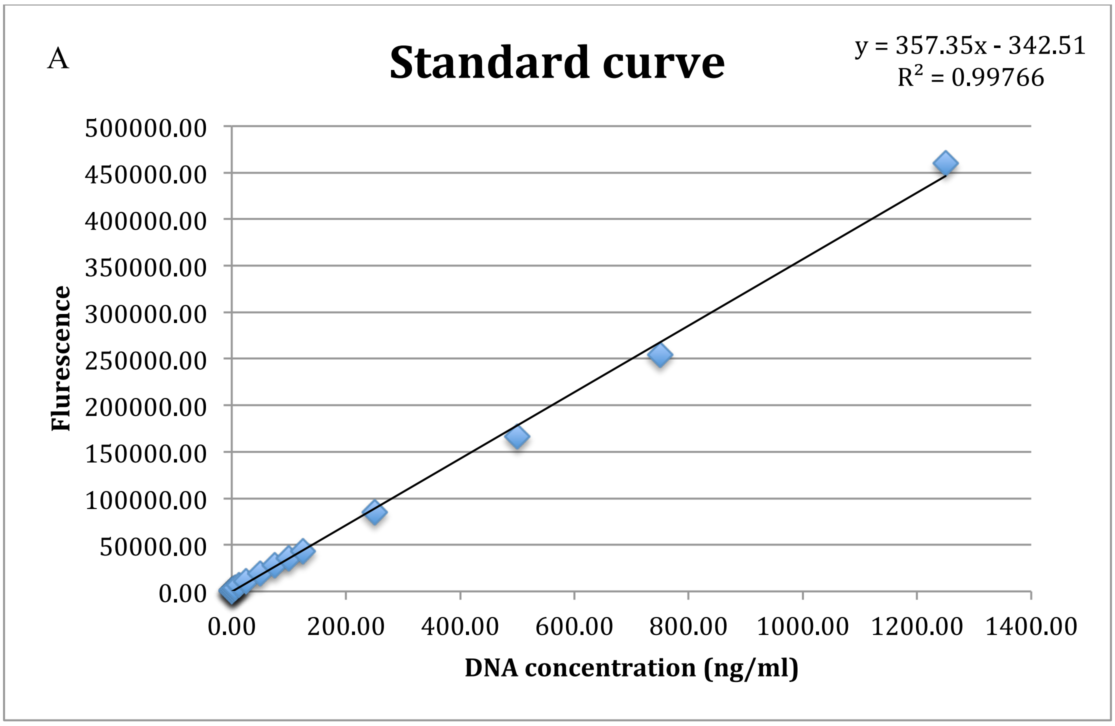

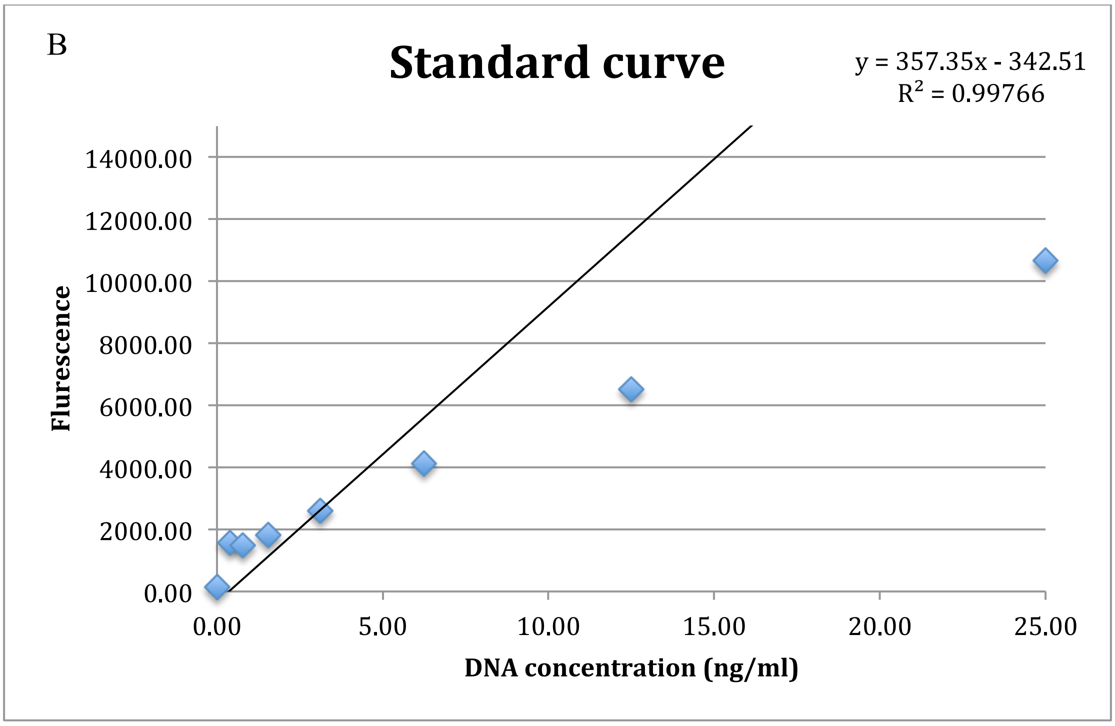

S1 Figure. Standard curve. (A) Complete standard curve, measured with fluorescence assay. (B) Standard curve magnified for higher dilutions.
