# Supplementary material for: Quantification of circulating cell-free DNA (cfDNA) in urine using a newborn piglet model of asphyxia
Source: PLoS One. 2019 Dec 31;14(12):e0227066. doi: 10.1371/journal.pone.0227066 (PMC6938324; doi:10.1371/journal.pone.0227066)
Supplement: S2 Fig — (A.) 50kb DNA ladder. Samples extracted by the direct method (E.) showed high-MW DNA contamination in comparison to samples extracted using the magnetic beads DNA extraction method (D.), where no high- or low-MW DNA was visible. Spiking the samples, adding non-fragmented human DNA (F.) (990 μl plasma spiked with 10 μl 1,250 ng/ml Human Genomic male DNA (Promega, Madison, USA) to the samples. Again, left over's of high-MW DNA was detected in samples gained by the direct method (C.), and no high- or low-MW DNA was visible in spiked samples processed with indirect method (B.). (PDF) [file pone.0227066.s002.pdf]

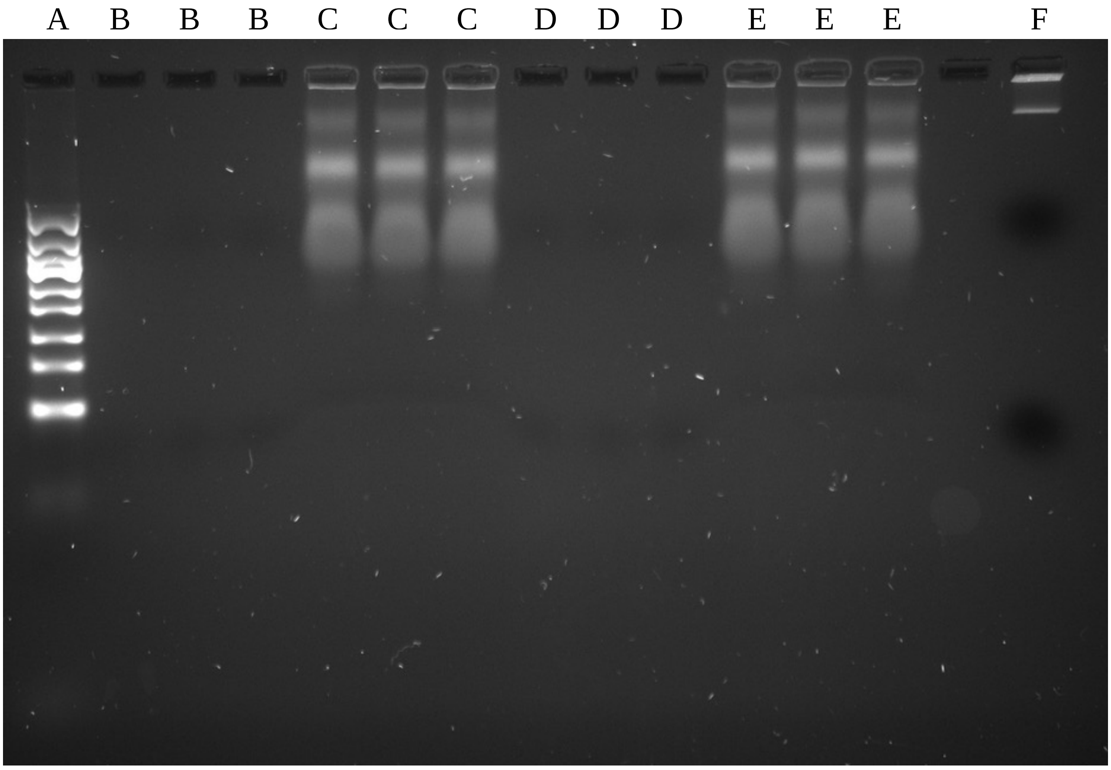

S2 Figure. Gel electrophoresis of spiked and not-spiked samples, processed with direct and indirect method. (A) 50kb DNA ladder. Samples extracted by the direct method (E) showed high-MW DNA contamination in comparison to samples extracted using the magnetic beads DNA extraction method (D), where no high- or low-MW DNA was visible. Spiking the samples, adding non-fragmented human DNA (F) (990 µl plasma spiked with 10 µl 1250 ng/ml Human Genomic male DNA (Promega, Madison, USA) to the samples. Again, left over's of high-MW DNA was detected in samples gained by the direct method (C), and no high- or low-MW DNA was visible in spiked samples processed with indirect method (B).
