# Supplementary material for: Quantification of circulating cell-free DNA (cfDNA) in urine using a newborn piglet model of asphyxia
Source: PLoS One. 2019 Dec 31;14(12):e0227066. doi: 10.1371/journal.pone.0227066 (PMC6938324; doi:10.1371/journal.pone.0227066)
Supplement: S1 Table — CfDNA levels in three therapy groups obtained using different methods. (PDF) [file pone.0227066.s003.pdf]

	Mean (S.D.)<br>Range (min - max)		
**Method**	**Hypoxia (n=6)**	**Hypoxia + hypothermia (n=6)**	**Controls (n=6)**
Flurescence_direct	230.48 (142.25)	357.29 (158.94)	223.01 (69.23)
	132.43 – 501.95	149.04 – 581.46	132.55 – 205.24
Flurescence_indirect	1.23 (1.76)	4.47 (6.15)	2.75 (3.62)
	0.01 – 4.42	0.03 – 13.33	0.42 – 9.17
mDNA_direct	0.03 (0.07)	0.03 (0.04)	0.15 (0.19)
	0.00 – 0.17	0.00 – 0.08	0.00 – 0.37
mDNA_indirect	0.01 (0.01)	0.10 (0.12)	0.03 (0.04)
	0.00 – 0.02	0.00 – 0.22	0.00 – 0.10
gDNA_direct	0.21 (0.30)	0.14 (.)	0.19 (0.17)
	0.01 - 0.65	0.14	0.02 – 0.35
gDNA_indirect	0.13 (0.10)	0.58 (0.66)	0.19 (0.09)
	0.05 – 0.26	0.01 – 1.29	0.04 – 0.27

S1 Table. Descriptive statistics. CfDNA levels in three therapy groups, obtained using different methods.
