# Supplementary material for: Quantification of circulating cell-free DNA (cfDNA) in urine using a newborn piglet model of asphyxia
Source: PLoS One. 2019 Dec 31;14(12):e0227066. doi: 10.1371/journal.pone.0227066 (PMC6938324; doi:10.1371/journal.pone.0227066)
Supplement: S2 Table — We found no recognizable pattern or statistically significant differences between the groups. (PDF) [file pone.0227066.s004.pdf]

Therapeutic group	Method	Ratio gDNA/mDNA
Hypoxia	Direct	6.42
	Indirect	22.10
Hypoxia and Hypothermia	Direct	5.37
	Indirect	5.65
Control	Direct	1.23
	Indirect	7.38

S2 Table. Ratio between mDNA and gDNA. We found no recognizable pattern or statistically significant differences between the therapeutic groups.
